# Beta‐Thalassemia in Spain: Results From the National Thalassemia Registry and Molecular Analysis of Patients With Transfusion‐Dependent Thalassemia

**DOI:** 10.1002/jcla.70272

**Published:** 2026-06-02

**Authors:** Ana Villegas, Paloma Ropero, Fernando Ataulfo González, Marta Morado, María Menor, Silvia de la Iglesia, Montserrat López‐Rubio, Eduardo José Salido, Mariola Abío, María Leonor Senent, Valle Recasens, José Manuel Vagace, Cristina Fonte, Jesús María Hernández‐Rivas, José María Raya, Pablo Ríos, Valeria Luciana Peri, Keneth A. Herrera, María Concepción Tenorio, Laura Lo Riso, Juan Tena, María Angustias Molina, Miriam Vara, Isabel Rodríguez, Pilar Ricard, Miguel Gómez, Oana Popa Dusacovschi, Julia María Vidán, Jorge Groiss, Óscar Ferre, Irene Orbe, Rosa María López, Nazaret Ugalde, Manuela Inés Hidalgo, Cristina Hinojosa, Ana Isabel Rodríguez, Esther Herrera, María Josefa Murúzabal, María del Carmen Hernández, Josefa Esperanza Marco, Celina Benavente, Grupo Español de Eritropatología

**Affiliations:** ^1^ Faculty of Medicine Universidad Complutense de Madrid Madrid Spain; ^2^ Instituto de Investigación Sanitaria San Carlos (IdISSC) Hospital Clínico San Carlos Madrid Spain; ^3^ Sociedad Española de Hematología y Hemoterapia Madrid Spain; ^4^ Hospital Universitario Príncipe de Asturias Madrid Spain; ^5^ Hospital Universitario de Gran Canaria Doctor Negrín Las Palmas de Gran Canaria Spain; ^6^ Hospital Clínico Universitario Virgen de la Arrixaca Murcia Spain; ^7^ Hospital General Universitario de Toledo Toledo Spain; ^8^ Hospital Universitario y Politécnico La Fe Valencia Valencia Spain; ^9^ Hospital Universitario Miguel Servet Zaragoza Spain; ^10^ Complejo Hospitalario de Badajoz Badajoz Spain; ^11^ Complejo Hospitalario de Vigo Pontevedra Spain; ^12^ Instituto de Investigación Biomédica de Salamanca Salamanca Spain; ^13^ Hospital Universitario de Canarias La Laguna Spain; ^14^ Hospital Nuestra Señora de Candelaria Santa Cruz de Tenerife Spain; ^15^ Hospital Universitario Insular de Gran Canaria Las Palmas de Gran Canaria Spain; ^16^ Hospital Clínico Universitario de Valladolid Valladolid Spain; ^17^ Hospital Universitario Ramón y Cajal Madrid Spain; ^18^ Hospital Universitario Son Espases Palma de Mallorca Spain; ^19^ Hospital General Mateu Orfila Menorca Spain; ^20^ Hospital Universitario Poniente Almería Spain; ^21^ Hospital Universitario de Cruces Bilbao Spain; ^22^ Hospital Universitario Puerta del Mar Cádiz Spain; ^23^ Hospital Universitario Fundación Alcorcón Madrid Spain; ^24^ Hospital Clínico San Carlos Madrid Spain; ^25^ Hospital Universitario de León León Spain; ^26^ Hospital Universitario de Badajoz Badajoz Spain; ^27^ Hospital Campo Arañuelo Navalmoral de la Mata Spain; ^28^ Hospital Virgen del Puerto Plasencia Spain; ^29^ Hospital Universitario de Cáceres Cáceres Spain; ^30^ Hospital General de Llerena Llerena Spain; ^31^ Hospital de Mérida Mérida Spain; ^32^ Hospital Materno‐Infantil Hospital Regional Universitario de Málaga Málaga Spain; ^33^ Hospital Universitario Juan Ramón Jiménez Huelva Spain; ^34^ Hospital Universitario Marqués de Valdecilla Santander Spain; ^35^ Hospital Don Benito–Villanueva de la Serena Don Benito Spain; ^36^ Hospital Universitario Doctor Peset Valencia Spain

**Keywords:** beta‐thalassemia, HBB mutations, molecular genetics, national registry, transfusion‐dependent thalassemia

## Abstract

**Background:**

Beta‐thalassemia is a genetically heterogeneous hemoglobinopathy with marked clinical variability. In Spain, comprehensive nationwide data on transfusion‐dependent beta‐thalassemia (TDT) remain limited, particularly regarding molecular characterization.

**Methods:**

This observational study analyzed data from the National Thalassemia Registry of the Spanish Society of Hematology and Hemotherapy (SEHH). Between November 2022 and February 2025, 147 patients with beta‐thalassemia from 42 hospitals were registered; this report focuses on 78 patients with TDT. Clinical, transfusion, and biochemical data were collected, and molecular analysis of the *HBB* and *HBA* genes was performed using Sanger sequencing, next‐generation sequencing, and complementary techniques. Genotypes were classified according to the degree of beta‐globin synthesis reduction.

**Results:**

The mean age of TDT patients was 34.3 years, and 73.1% were of Spanish origin. Patients received a mean of 31.4 packed red blood cell units per year. Splenectomy had been performed in 35.9% of cases. Most patients showed adequate iron overload control, with median serum ferritin levels below 1000 ng/mL. Twenty‐four different *HBB* mutations were identified; the most frequent were CD39 (C > T), IVS‐1‐nt1 (G > A), IVS‐1‐nt110 (G > A), IVS‐1‐nt6 (T > C), and IVS‐1‐nt1 (G > T), accounting for 75% of alleles. Genotype distribution was 55.2% β^0^/β^0^, 30.3% β^0^/β^+^, and 14.4% β^+^/β^+^. Patients with β^+^/β^+^ genotypes had significantly lower ferritin levels.

**Conclusions:**

This nationwide registry highlights the genetic and clinical heterogeneity of TDT in Spain and underscores the value of molecular characterization for patient management, genetic counseling, and future therapeutic strategies.

## Graphical abstract

This nationwide registry‐based study describes the clinical and molecular characteristics of 78 patients with transfusion‐dependent beta‐thalassemia in Spain. Comprehensive genetic analysis revealed marked molecular heterogeneity, with 24 *HBB* mutations identified and a predominance of β^0^ genotypes, alongside generally effective transfusion and iron chelation management. These data highlight the value of national registries and molecular characterization to support clinical care, genetic counseling, and future therapeutic strategies.

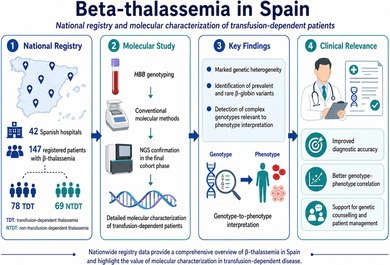

## 1 Introduction

Thalassemias are a heterogeneous group of hemoglobinopathies characterized by decreased or absent synthesis of one or more of the globin chains forming hemoglobin (Hb) [1]. They exhibit considerable genetic and clinical variability and are classified based on the affected chain, with alpha‐ and beta‐thalassemia occurring more frequently, since alpha‐ and beta‐globin chains form HbA, the predominant Hb in postnatal life [1, 2]. Some structural hemoglobinopathies are characterized by a deficiency in the synthesis of structurally abnormal hemoglobin molecules through various mechanisms. For example, Hb Lepore, a variant relatively common in Spain, also leads to a thalassemia phenotype with microcytosis and hypochromia.

Clinically, beta‐thalassemia is classified as follows: thalassemia major (the most severe form, requiring regular transfusions), thalassemia trait (asymptomatic carriers with microcytosis, hypochromia, and sometimes mild anemia), intermediate thalassemia (a highly variable group of symptomatic thalassemias, including cases with slightly more severe anemia than thalassemia trait, although transfusions are not required, as well as cases less severe than thalassemia major but still requiring occasional transfusions under biological stress or to prevent or treat complications of the disease), and silent thalassemia (so mild that there are no symptoms, microcytosis, or hypochromia). A distinction is made between transfusion‐dependent thalassemias (TDT) and non‐transfusion‐dependent thalassemias (NTDT). People with NTDT do not require regular transfusions and may only need them on rare occasions, if ever [2, 3].

This study describes cases included in the National Thalassemia Registry of the Spanish Society of Hematology and Hemotherapy (SEHH—*Sociedad Española de Hematología y Hemoterapia*) over the last 28 months. Clinical, analytical, transfusion, and molecular genetic data were analyzed to ensure thorough documentation, facilitating the development of applications for future treatments and clinical trials.

## 2 Material and Methods

A total of 147 patients diagnosed with beta‐thalassemia from 42 hospitals across Spain were included in this observational study between November 2022 and February 2025. Of these, 78 were classified as transfusion‐dependent thalassemia (TDT) and 69 as non‐transfusion‐dependent thalassemia (NTDT). Classification as TDT was based on clinical criteria, including early presentation (≤ 2 years of age) and the requirement for regular transfusions to maintain adequate hemoglobin levels.

Clinical follow‐up data were collected using a standardized data collection form distributed to all participating centers. Although some variability in clinical practice may exist between hospitals, participating centers generally follow international guidelines for the management of transfusion‐dependent thalassemia. All cases were included in the National Thalassemia Registry of the Spanish Society of Hematology and Hemotherapy (SEHH), enabling the systematic collection of clinical, analytical, and molecular data from both native and migrant populations.

During the final eight months of the data collection period, eight newly diagnosed patients were identified. Although both TDT and NTDT patients were included in the registry, the present study focuses exclusively on TDT patients, as the statistical analysis of the NTDT cohort was not fully completed at the time of manuscript preparation. A separate analysis of NTDT patients is currently ongoing and will be reported in a future study.

For TDT patients, the following variables were analyzed: demographic characteristics (age, sex, and country of origin of patients and their parents), transfusion requirements over the previous year, history of splenectomy and age at surgery, current treatments (including iron chelation therapy and luspatercept), serum ferritin levels as a surrogate marker of iron overload, and comprehensive molecular characterization of the HBA and HBB genes.

Patients with structural hemoglobinopathies such as HbS, HbC, or HbD, as well as those with compound heterozygosity for beta‐thalassemia and structural variants were excluded from the study.

All patients provided written informed consent in accordance with the Declaration of Helsinki, and the study was approved by the corresponding institutional ethics committee.

### 2.1 Hematological and Biochemical Analysis

Hemoglobin variants were identified and quantified using two complementary techniques: high‐performance liquid chromatography (HPLC) with the VARIANT II system (Bio‐Rad Laboratories, Hercules, CA, USA) and capillary electrophoresis (CE) with the Minicap Flex‐Piercing system (Sebia, Norcross, GA, USA), following the manufacturers' instructions and previously validated protocols [4].

### 2.2 Molecular Analysis

Genomic DNA was extracted from peripheral blood using the automated EZ1 DNA Blood Kit (Qiagen GmbH, Hilden, Germany) and quantified using a Qubit 3 fluorometer (Thermo Fisher Scientific, Waltham, MA).

Alpha‐globin gene alterations were initially assessed using the α‐Globin StripAssay (ViennaLab Diagnostic GmbH, Vienna, Austria), which allows detection of the most common deletions and point mutations. In cases requiring further characterization, multiplex ligation‐dependent probe amplification (MLPA) was performed using SALSA MLPA probemix P140 HBA (MRC‐Holland, Amsterdam, The Netherlands).

Variants in the *HBB* gene were characterized by Sanger sequencing of all exons and promoter regions, following standardized protocols [5, 6].

During the last phase of the study, next‐generation sequencing was implemented in our laboratory as a routine diagnostic tool. Therefore, the last 33 patients included in the registry were analyzed using the Devyser Thalassemia kit (Devyser, Årsta, Sweden). This assay can detect single‐nucleotide variants, insertions and deletions, and copy number variations in *HBA1*, *HBA2*, and *HBB* genes. Libraries were prepared according to the manufacturer's instructions, and sequencing was performed on a MiSeq system (Illumina, San Diego, CA). Bioinformatic analysis was performed using Devyser Amplicon Suite v3.8 software [7].

### 2.3 Statistical Analysis

Statistical analyses were performed using IBM SPSS Statistics, version 27.0 (IBM Corp., Armonk, NY). Quantitative variables were expressed as mean ± standard deviation (SD) for normally distributed data, or as median and interquartile range (IQR) when normality was not met (assessed using the Kolmogorov–Smirnov test).

Categorical variables were expressed as absolute frequencies and percentages and compared using the chi‐squared (χ^2^) test or Fisher's exact test, as appropriate. Quantitative variables were compared using analysis of variance (ANOVA) when more than two groups were analyzed, Student's *t*‐test for independent samples, or the Mann–Whitney U test when data were not normally distributed. Correlations between continuous variables were assessed using Pearson or Spearman correlation coefficients, depending on data distribution.

A *p* < 0.05 was considered statistically significant.

## 3 Results

### 3.1 Cohort Characteristics

A total of 147 patients with a confirmed diagnosis of beta‐thalassemia were included in the registry, of whom 78 (53%) were classified as transfusion‐dependent thalassemia (TDT) and 69 (47%) as non‐transfusion‐dependent thalassemia (NTDT). All patients underwent comprehensive molecular characterization of the HBA and HBB genes. The present analysis focuses exclusively on the TDT subgroup.

The distribution of TDT patients across participating centers was heterogeneous, with only three hospitals contributing five or six cases, whereas most centers included one or two patients. No deaths were recorded during the study period.

Among the 78 TDT patients, 36 (46.2%) were female, and 42 (53.8%) were male. The mean age was 34.29 ± 16.85 years (interquartile range [IQR] 24.5). Twenty‐five patients were between 19 and 40 years of age, 39 were older than 40 years, and 14 were younger than 18 years. Five patients were diagnosed through newborn screening and evaluated during the first months of life; among these, only two had Spanish parents, and none had received prior genetic counseling.

Regarding geographical origin, 73.1% of patients were of Spanish origin, whereas 26.9% were foreign‐born or had foreign‐born parents. Among these, the most represented populations were individuals of Indian origin (10.25%), followed by North African (8.97%), Portuguese (2.56%), and other origins (5.12%).

### 3.2 Transfusion Burden and Clinical Management

The mean number of packed red blood cell (PRBC) units transfused during the previous year was 31.43 ± 12.20 (range 10–54). Splenectomy had been performed in 35.9% of patients at a mean age of 11.65 ± 4.12 years.

Mean serum ferritin levels were 992.42 ± 1573.89 ng/mL, with a median value of 487 ng/mL. Overall, 78% of patients had ferritin levels below 1000 ng/mL, suggesting generally adequate control of iron overload.

No significant differences were observed between male and female patients in terms of age, transfusion requirements, or age at splenectomy. Although ferritin levels were lower in males than in females (718.87 ± 701.28 vs. 1289.43 ± 2129.81 ng/mL), these differences did not reach statistical significance (Table 1).

**TABLE 1:** Clinical variables analyzed according to sex.

	All	Male	Female	*p*
Age years (*n*)	34.29 ± 16.85 (78)	34.82 ± 18.73 (42)	33.69 ± 14.64 (36)	0.77
Spanish % (*n*)	73.1 (57)	73.8 (31)	72.2 (26)	0.87
Number of transfusions (*n*)	31.43 ± 12.20 (67)	31.44 ± 11.9 (34)	31.42 ± 12,65 (33)	0.99
Splenectomized Patients % (*n*)	35.9 (28)	35.7 (15)	36.1 (13)	0.97
Age at splenectomy years (*n*)	11.65 ± 4.12 (17)	11.33 ± 4.0 (9)	12 ± 4.5 (8)	0.75
Ferritin ng/mL (*n*)	992.42 ± 1573.89 (73)	718.87 ± 701.28 (38)	1289.43 ± 2129.81 (35)	0.13

Splenectomized patients were significantly older than non‐splenectomized patients (43.78 ± 9.23 vs. 28.87 ± 17.86 years; *p* < 0.001). Although splenectomized patients showed lower ferritin levels (653.81 ± 571.21 vs. 1191.17 ± 1914.61 ng/mL), this difference was not statistically significant. Similarly, no significant differences were observed in transfusion requirements between the two groups (Table 2).

**TABLE 2:** Clinical variables analyzed according to splenectomy.

	All	Splenectomized	No splenectomized	*p*
Sex male % (*n*)	53.8 (42)	53 (15)	54 (27)	0.97
Age years (*n*)	34.29 ± 16,85 (77)	43.78 ± 9,23 (28)	28.87 ± 17.86 (49)	< 0.001
Spanish % (*n*)	73.1 (57)	78.6 (22)	70 (35)	0.41
Number of transfusions (*n*)	31.43 ± 12.20 (67)	34.25 ± 11.27 (28)	29.41 ± 12.58 (39)	0.11
Ferritin ng/mL (*n*)	992.42 ± 1573.89 (73)	653.81 ± 571.21 (27)	1191.17 ± 1914.61 (46)	0.081

A significant positive correlation was identified between patient age and the number of transfusions received (*r* = 0.29, *p* = 0.019), as well as between serum ferritin levels and transfusion burden (*r* = 0.34, *p* = 0.023). No correlation was observed between age and ferritin levels.

### 3.3 Iron Chelation Therapy and Additional Treatments

Deferasirox (DFX) was the most frequently used iron chelation therapy, administered to 61 patients. A small number of patients required alternative or combined treatments: three patients received deferiprone (DFP) alone, four received deferoxamine (DFO) alone, four received combined DFP and DFX therapy, and one received combined DFP and DFO therapy. Six patients were not receiving treatment due to age below 2 years, and treatment data were unavailable for four patients.

Significant differences in serum ferritin levels were observed between different chelation regimens (*p* < 0.001), with the highest levels observed in patients receiving combination therapy. No significant differences were found between treatment groups in terms of age, transfusion burden, or age at splenectomy (Table 3).

**TABLE 3:** Clinical variables analyzed according to chelation therapies.

	All	DFX	DFP	DFO	Combined	*p*
Sex male % (*n*)	52.9 (36)	57.1 (32)	33.3 (1)	75 (3)	0	0.06
Age (years)	37.89 ± 13.19 (68)	38.78 ± 13.44 (54)	43.33 ± 10.69 (3)	29.5 ± 13.57 (5)	31.6 ± 8.84 (4)	0.31
Spanish % (*n*)	79.4 (54)	76.8 (43)	66.7 (2)	100 (4)	100 (5)	0.41
Number of transfusions (*n*)	31.73 ± 12.05 (66)	31.20 ± 11.07 (56)	27.67 ± 20.40 (3)	24.00 ± 11.31 (2)	43.2 ± 14.93 (5)	0.12
Splenectomized patients (%)	41.2 (28)	42.9 (24)	66.7 (2)	25 (1)	20 (1)	0.52
Age at splenectomy years (*n*)	11.65 ± 4.12 (17)	10.87 ± 3.6 (15)	20 (1)		15 (1)	0.06
Ferritin ng/mL (*n*)	1110.78 ± 1647.83 (64)	798.61 ± 936.37 (52)	553 ± 633.41 (3)	707 ± 8.48 (2)	4978.4 ± 3258.96 (5)	< 0.001

Fifteen patients received luspatercept at a maximum dose of 1.5 mg/kg every 21 days, of whom five (33.3%) achieved a clinical response defined as an increase in hemoglobin levels of ≥ 1.5 g/dL. One patient achieved transfusion independence [8]. Three additional patients had recently initiated treatment, precluding evaluation of response.

Four patients underwent allogeneic hematopoietic stem cell transplantation from HLA‐identical sibling donors. In these cases, sustained engraftment with complete chimerism and good immunological tolerance was observed.

### 3.4 Molecular Characterization

A total of 24 different mutations in the HBB gene were identified (Table 4). The most frequent variants were CD39 (C > T), IVS‐1‐nt1 (G > A), IVS‐1‐nt6 (T > C), IVS‐1‐nt110 (G > A), and IVS‐1‐nt1 (G > T), accounting for approximately 75% of all alleles.

**TABLE 4:** HBB mutations registered in the cohort of patients with transfusion‐dependent β‐thalassemia (TDT).

Classical nomenclature	HGVS nomenclature	Allelic phenotype	Homozygous state	Heterozygous state	Total mutated alleles	Allele frequency (%)
Codon 39 (C > T)	HBB: c.118C > T	_β_0	12	17	41	26.6
IVS‐I‐1 (G > A)	HBB: c.92 + 1G > A	_β_+	9	9	27	17.5
IVS‐I‐6 (T > C)	HBB: c.92 + 6 T > C	_β_+	5	10	20	13
IVS‐I‐110 (G > A)	HBB: c.93‐21G > A	_β_0	2	12	16	10.4
IVS‐I‐1 (G > T)	HBB: c.92 + 1G > T	_β_+	3	7	13	8.4
Codon 6 (−A)	HBB: c.20delA	_β_0	0	4	4	2.6
Codon 8 (−AA)	HBB: c.25_26delAA	_β_0	0	4	4	2.6
619 bp deletion	NG_000007.3: g.71609_72227del619	_β_0	0	4	4	2.6
IVS‐II‐848 (C > A)	HBB: c.316‐3C > A	_β_+	0	4	4	2.6
Codons 41/42 (‐TTCT)	HBB: c.126_129delCTTT	_β_0	1	1	3	1.9
Spanish (deltabeta)^0^‐Thal	NG_000011.10: g.5144331_5237241del	_β_0	0	3	3	1.9
IVS‐I‐2 (T > G)	HBB: c.92 + 2 T > G	_β_0	0	2	2	1.3
−28 (A > G)	HBB: c.‐78 A > C	_β_+	0	2	2	1.3
Poly A (A > G) AATAAA > AATGAA	HBB: c.*111A > G	_β_+	0	1	1	0.6
IVS‐I‐5 (G > C)	HBB: c.92 + 5G > C	_β_+	0	1	1	0.6
IVS‐II‐2 (T > G)	HBB: c.315 + 2 T > G	_β_0	0	1	1	0.6
IVS‐II‐745 (C > G)	HBB: c.316–106 C > G	_β_+	0	1	1	0.6
Codon 11 (−T)	HBB: c.36delT	_β_0	0	1	1	0.6
Codons 82/83 (−G)	HBB: c.251delG	_β_0	0	1	1	0.6
Hb Knossos	HBB: c.82G > T	_β_+	0	1	1	0.6
Hb Monroe	HBB: c.92G > C	_β_0	0	1	1	0.6
Codon 10 (GCC > GCA)	HBB: c.33C > A	_β_+	0	1	1	0.6
Codons 8/9 (+ G)	HBB: c.27_28insG	_β_0	0	1	1	0.6
Codon 16 (GGC > GG‐)	HBB: c.51delC	_β_0	0	1	1	0.6
TOTAL			32	90	154	

Abbreviation: HGSV, Human Genome Variation Society.

CD39 (C > T) was the most prevalent mutation, identified in 29 patients, including 12 homozygous cases. IVS‐1‐nt1 (G > A) was detected in 18 patients, eight of whom were homozygous. IVS‐1‐nt6 (T > C) was identified in 15 patients, while IVS‐1‐nt110 (G > A) and IVS‐1‐nt1 (G > T) were observed in 14 and 18 patients, respectively.

Less frequent mutations included CD8/9 (+G) mutation, CD8 (−AA), Poly A (A > G), and the 619‐bp deletion, among others. These variants were typically found in compound heterozygosity with more common mutations.

In addition to beta‐globin gene alterations, two patients presented homozygous alpha‐globin gene triplication (αααanti3.7/αααanti3.7), and three patients showed alpha‐globin gene deletions (−α3.7/αα). Three cases of delta‐beta thalassemia were also identified.

### 3.5 Genotype Distribution and Phenotype Correlation

The most frequent complete HBB genotypes are summarized in Table 5. Homozygous CD39 (C > T) and IVS‐1‐nt1 (G > A) genotypes were the most common, while the remaining cases consisted of multiple compound heterozygous combinations.

**TABLE 5:** Frequency of complete HBB genotypes in patients with transfusion‐dependent β‐thalassemia.

Genotype	Number of patients	Frequency (%)
CD39 (C > T) / CD39 (C > T)	12	15.4
IVS‐1‐nt1 (G > A) / IVS‐1‐nt1 (G > A)	8	10.3
IVS‐1‐nt6 (T > C) / IVS‐1‐nt6 (T > C)	5	6.4
IVS‐1‐nt110 (G > A) / IVS‐1‐nt110 (G > A)	2	2.6
IVS‐1‐nt1 (G > T) / IVS‐1‐nt1 (G > T)	3	3.8
CD39 (C > T) / IVS‐1‐nt1 (G > A)	4	5.1
CD39 (C > T) / IVS‐1‐nt6 (T > C)	3	3.8
CD39 (C > T) / IVS‐1‐nt110 (G > A)	2	2.6
IVS‐1‐nt1 (G > A) / IVS‐1‐nt6 (T > C)	2	2.6
IVS‐1‐nt110 (G > A) / CD39 (C > T)	2	2.6
Other rare compound genotypes	35	44.9
Total	**78**	**100**

Patients were classified according to the severity of beta‐globin synthesis impairment into β0/β0, β0/β+, and β+/β + genotypes. Two patients with alpha‐globin gene triplication were excluded from this classification. Among the remaining patients, 42 (55.2%) had a β0/β0 genotype, 23 (30.3%) had β0/β+, and 11 (14.4%) had β+/β + .

Significant differences were observed in serum ferritin levels among genotype groups (*p* = 0.004), with β+/β + patients showing markedly lower ferritin levels compared with β0/β0 and β0/β + patients. No statistically significant differences were observed between genotypes in terms of age, transfusion burden, age at splenectomy, sex distribution, or geographical origin (Table 6).

**TABLE 6:** Clinical variables analyzed according to genotype.

	All	β0/β0	β0/β+	β+/β+	*p*
Sex male % (*n*)	52.6 (40)	61.9 (26)	34.8 (8)	54.5 (6)	0.11
Age (years)	34.42 ± 16.86 (75)	32.09 ± 17.11 (41)	34.13 ± 16 (23)	43.72 ± 15.87 (11)	0.19
Spanish % (*n*)	72.4 (55)	64.3 (27)	91.3 (21)	63.7 (7)	0.52
Number of transfusions (*n*)	31.43 ± 12.2 (67)	32.03 ± 11.95 (36)	32.38 ± 13.02 (21)	27.3 ± 11.71 (10)	0.51
Splenectomized patients (%)	36.8 (28)	31 (13)	39.1 (9)	54.5 (6)	0.34
Age at splenectomy years (*n*)	11.65 ± 4.12 (17)	11.33 ± 4.27 (6)	11.5 ± 4.41 (6)	12.2 ± 4.49 (5)	0.94
Ferritin ng/mL (*n*)	992.42 ± 1573.89 (73)	1004.95 ± 1652.12 (41)	1322.43 ± 1754.02 (21)	315.73 ± 176.90 (11)	0.004

## 4 Discussion

Thalassemias are among the most common monogenic diseases worldwide, with approximately 300,000–400,000 affected births annually, including 40,000–50,000 TDT cases [9], mainly in regions from the Mediterranean to Indonesia and parts of tropical and subtropical Africa, with a high incidence of heterozygotes in Italy, Cyprus, and Greece. Due to migration, cases now also occur across Europe and America [10].

The incidence of thalassemia in Spain has historically been much lower than that in Greece or Italy. Mild or heterozygous forms occur in 0.1%–2% of the population [11]. Severe thalassemias are much less common in Spain than in Italy or Greece. Due to the implementation of premarital and prenatal programs by the Spanish Red Blood Cell Pathology Group of the SEHH, the number of children born in Spain to Spanish parents and ancestors is very low. In this study, nearly a quarter of patients were foreign‐born or children born in Spain to foreign parents. In the last 28 months, five cases were diagnosed through neonatal screening, mostly of foreign origin, and two children of Spanish parents and families had not received genetic counseling. This highlights the importance of including immigrant populations in the Spanish health system and strengthening premarital and prenatal programs to prevent the birth of children with severe thalassemia. Although correlations between genotype and phenotype are unpredictable, molecular characterization can help support appropriate genetic counseling.

In Spain, carrier screening and genetic counseling programs have been progressively implemented across the autonomous communities and have contributed to reducing the number of new TDT cases among families of Spanish origin. Among the 18 patients with TDT aged ≤ 20 years included in the cohort, only three had both parents of Spanish origin. Two additional cases belonged to families from specific ethnic backgrounds in which adherence to genetic counseling recommendations was limited despite prior counseling, and recent migration patterns have introduced additional genetic diversity into the population, highlighting the need to reinforce culturally adapted prevention strategies. These observations emphasize the importance of strengthening culturally adapted screening and prevention strategies.

Based on estimates from previous national studies and clinical experience, the total number of patients with transfusion‐dependent β‐thalassemia currently diagnosed in Spain is estimated to be around 100 cases. Therefore, the 78 patients included in the present study likely represent a substantial proportion of the national TDT population.

The mean age of TDT patients was 35 years, with only 17.7% being younger than 18 years. According to the Spanish Registry of the Spanish Society of Pediatric Hematology and Oncology (Sociedad Española de Hematología y Oncología Pediátrica—SEHOP) [12], 75% of patients were pediatric. Over the last few decades, the development, dissemination, and widespread use of iron chelation therapy, together with improved transfusion regimens and treatments for various complications of the disease [13, 14, 15], have improved survival. In our case series, 47,5% of patients were over 40 years of age, and no deaths were recorded during the study period. Foreign patients were younger than Spanish patients, likely reflecting selection bias due to the higher number of births to foreign parents and immigration constraints for individuals with serious, chronic illnesses.

All TDT patients received transfusions, with a mean of 31.43 units of PRBCs in the previous year to maintain Hb levels > 9 g/dL. As expected, a significant correlation was found between the number of transfusions in the previous year and patient age due to the inclusion of pediatric patients in this registry. For this reason, foreign patients received fewer transfusions as they were younger.

Historically, most patients with transfusion‐dependent β‐thalassemia in Spain were treated with deferoxamine. In recent years, oral chelation therapy with deferasirox has become the most commonly used first‐line treatment due to its ease of administration and favorable tolerability profile. Deferiprone is generally reserved for cases of insufficient response or severe iron overload, and combination therapy may be considered in selected patients. Patients receiving DFP had lower serum ferritin levels than those receiving DFX and DFO, potentially reflecting selection bias, as most patients were treated with DFX due to its more convenient dosage and better tolerance, and its introduction in Spain followed that of DFP. Therefore, many patients may have switched from DFP to DFX, and only a few patients who showed a very good response to DFP would have continued the treatment. Similarly, patients on combination therapy had significantly higher serum ferritin levels than those on monotherapy, which likely represented the indication for combination treatment.

Although magnetic resonance imaging of the liver and heart is currently considered the best method for iron overload estimation, due to access limitations in many hospitals, serum ferritin levels were assessed. Most patients received chelation therapy with good control of iron overload, maintaining serum ferritin levels < 1000 μg/dL.

Regarding splenectomy, its indications in TDT have become more restrictive due to associated complications such as thrombosis and pulmonary hypertension [10, 13]. In our case series, splenectomized patients were significantly older than non‐splenectomized patients, with a mean age at splenectomy of 11 years, indicating that the number of splenectomies has decreased over recent years. Although the main indication for splenectomy is to reduce the number of annual transfusions, no significant differences were found in the number of units transfused per year between splenectomized and non‐splenectomized patients. The relatively small number of splenectomized patients limited the possibility of performing a more detailed stratified analysis according to age groups. However, since age was associated with the number of transfusions and splenectomized patients were significantly older, it is difficult to assess whether splenectomy reduced transfusion requirements in our case series. In this regard, splenectomized patients had lower serum ferritin levels than non‐splenectomized patients. As serum ferritin levels and the number of transfusions were significantly correlated, the lower ferritin levels in splenectomized patients could potentially be related to a reduced number of transfusions. However, other causes, such as less ineffective extramedullary erythropoiesis, could also contribute to the lower ferritin levels in splenectomized patients.

Other complications of the disease, such as endocrine disorders, osteopenia/osteoporosis, alloimmunization, kidney disease, cardiac disease, cholelithiasis, pulmonary hypertension, thrombosis, stroke, and viral infections including hepatitis B, hepatitis C, and HIV, were not included in this initial analysis of the registry. We plan to incorporate these variables in future updates in order to provide a more comprehensive assessment of the disease burden. Although virological screening is routinely performed in clinical practice in Spain, these parameters were not included in the initial data collection of the registry.

As for molecular lesions, the *HBB* and *HBA* alleles were analyzed in 78 TDT patients. The most frequent mutation was CD39 (C > T), followed by IVS‐1‐nt1 (G > A), IVS‐1‐nt6 (T > C), IVS‐1‐nt110 (G > A), and IVS‐1‐nt1 (G > T), accounting for 75% of all mutations. Mutations were identified in all patients; of the 156 alleles analyzed, 154 carried pathogenic variants. These mutations are also found in other Mediterranean countries and in France, with similar percentages [10, 11, 16].

However, the present registry also reflects recent demographic changes in Spain. In particular, the increasing contribution of patients originating from South Asia, North Africa, and Eastern Europe has introduced additional genetic diversity into the molecular landscape of β‐thalassemia in the country. These findings highlight the influence of migration patterns on the epidemiology of hemoglobinopathies in Europe.

Our results corroborate previous studies carried out by our group, in which we found these mutations in 86.6% of the cases studied. However, in this new study, we identified only isolated cases of the CD8/9 (+G) mutation [11].

In terms of genotype, β+/β + patients had significantly lower serum ferritin levels and were older, which could be related to a less severe phenotype compared to β0/β0 and β0/β + patients, although other disease complications were not assessed in this study.

Within the β+/β + category, five patients were homozygous for the IVS‐1‐nt6 (T > C) mutation, which has been described as a thalassemia with a highly variable phenotype, including cases exhibiting NTDT behavior. Two of these patients were siblings followed at our hospital who were diagnosed early in childhood, before molecular characterization was routinely available. Because they required regular transfusions from an early age, they were initially managed as TDT patients. When molecular studies later revealed homozygosity for the IVS‐1‐nt6 mutation, transfusions were temporarily discontinued to evaluate their clinical phenotype. However, hemoglobin levels declined rapidly, and regular transfusion therapy had to be resumed, confirming a transfusion‐dependent phenotype. These observations illustrate the phenotypic variability associated with this mutation. In our case series, these five patients showed no significant differences in the variables studied compared to the rest of the β+/β + patients. Two patients, aged 7 and 45 years, were homozygous for the alpha‐globin gene triplication (ααα/ααα) and heterozygous for a β0 mutation [CD39 (C > T) and CD82/83 (−G)]. Both presented with a TDT phenotype, highlighting the possible influence of secondary molecular modulators, such as the degree of alpha‐globin synthesis, on the alpha/beta globin imbalance and, consequently, the severity of the disease [3, 4, 13, 17, 18]. Furthermore, another factor that may influence the severity of beta‐thalassemia is the lack of alpha‐globin detoxification by the chaperone alpha‐hemoglobin‐stabilizing protein (AHSP) [3]. Autophagy of these alpha‐globin chains also plays a fundamental role in phenotypic expression. The gene *AMBRA1* [19], involved in the clearance of toxic free alpha‐globin chains, has been studied in thalassemia, both in animal models and humans. Mutations in *AMBRA1* may impair alpha‐globin clearance, significantly influencing the thalassemic phenotype.

This study demonstrated the high variability in the genotype and phenotype of TDT patients in Spain. The main limitations of registry studies are the lack of standardized follow‐up protocols across participating hospitals and the possibility that not all patients with the disease have been registered. In our study, the 78 patients were followed up at 42 different hospitals; therefore, bias in the interpretation of the results is a concern. However, this has enabled us to establish a new registry for developing more in‐depth analytical studies, planning future treatments and potential clinical trials, and identifying at‐risk groups with unmet healthcare needs.

In conclusion, this study provides an updated overview of the clinical and molecular characteristics of patients with transfusion‐dependent β‐thalassemia included in the Spanish National Thalassemia Registry. The data highlight the genetic heterogeneity of the disease and reflect recent demographic changes influencing the epidemiology of β‐thalassemia in Spain. Molecular characterization of these patients is essential to facilitate appropriate genetic counseling and to identify candidates for emerging therapeutic strategies such as gene therapy and other innovative treatments.

## Funding

This work was partly supported by a grant from Agios Pharmaceuticals.

## Ethics Statement

This study was conducted in accordance with the Declaration of Helsinki and was approved by the appropriate institutional ethics committee.

## Consent

Written informed consent was obtained from all participants included in the study.

## Conflicts of Interest

The authors declare no conflicts of interest.

## Data Availability

The data that support the findings of this study are available from the corresponding author upon reasonable request.
